# Adherence to Carbohydrate Counting Improved Diet Quality of Adults with Type 1 Diabetes Mellitus during Social Distancing Due to COVID-19

**DOI:** 10.3390/ijerph19169776

**Published:** 2022-08-09

**Authors:** Gabriela Correia Uliana, Manuela Maria De Lima Carvalhal, Talita Nogueira Berino, Aline Leão Reis, Karem Miléo Felício, João Soares Felício, Daniela Lopes Gomes

**Affiliations:** 1 Postgraduate Program in Neurosciences and Behavior, Nucleus of Behavior Theory Research, Federal University of Pará, Belém 66075-110, Brazil; 2 Faculty of Nutrition, Federal University of Pará, Belém 66075-110, Brazil; 3 Postgraduate Program in Attention and Clinical Study in Diabetes, Institute of Health Sciences, Federal University of Pará, Belém 66075-110, Brazil

**Keywords:** diabetes mellitus, carbohydrates, COVID-19, social distancing

## Abstract

To control glycemic variability in people with Type 1 diabetes mellitus (T1DM), it is essential to perform carbohydrate counting (CC), a strategy that ensures better quality of life for these patients. Thus, this study aims to analyze potential factors associated with adherence to CC in adults with T1DM during social distancing due to COVID-19 in Brazil. This was a single cross-sectional study carried out in July 2020. An online form was used to collect sociodemographic and economic data on the purchasing of supplies and food, as well as social distancing. The Chi-square test was performed with adjusted residuals analysis and a binomial logistic regression analysis (*p* < 0.05). Of 472 adults, 37.71% reported performing CC in the same frequency as before social distancing. There was an association between performance of CC and the type of city (*p* = 0.027), family income (*p* = 0.000), use of financial emergency aid (*p* = 0.045), type of insulin administration and glycemic monitoring (*p* < 0.000), and cooking more (*p* = 0.012). Participants who maintained or reduced consumption of ultra-processed foods were 0.62 times more likely to adhere to CC (OR 0.626, 95% IC: 0.419–0.935) and participants who cooked more were 1.67 times more likely to adhere to CC (OR 1.67, 95% CI: 1.146–2.447). There are still people with T1DM who did not know about and did not use CC method, which highlights the need for diabetes education.

## 1. Introduction

COVID-19 was identified for the first time in December 2019 in the city of Wuhan, China, and is a severe acute respiratory syndrome caused by Coronavirus-2 (SARS-CoV-2) [1]. In Brazil, COVID-19 was declared a Public Health Emergency (PHE) in February 2020, and from that point on, Law No. 13.979 was approved, with measures to cope with COVID-19 [2,3].

People with Type 1 diabetes mellitus (T1DM) require special treatment during the current COVID-19 pandemic, as studies show that people with T1DM are at increased risk of infections and hospitalizations compared to persons who do not have diabetes [4,5]. According to Ebekozien et al. [6], more than 50% of people with T1DM who had or were suspected to have had COVID-19 reported hyperglycemia as one of the symptoms. Thus, it is necessary to identify a regular treatment to maintain stable glycemia during the pandemic [7].

One of the most important aspects of glycemic control and the prevention of complications is the adoption of a healthy eating pattern, prioritizing the ingestion of fresh or minimally processed foods [7,8]. To control glycemic variability, it is important to perform carbohydrate counting (CC), a strategy that ensures better quality of life for people with T1DM, as it helps in glycemic control and allows greater flexibility in eating [7,9]. This strategy promotes the balance between the glycemia’s value, the amount of ingested carbohydrates, and the amount of insulin that should be applied [7]. A meta-analysis carried out by Fu et al. [10] showed that CC, when compared to other dietary methods or the usual diet for diabetics, significantly reduces the concentration of glycated hemoglobin and indicated that CC should be recommended for the routine treatment of patients with T1DM.

For the implementation and efficiency of CC, it is necessary that the patient regularly measures glycemia and insulin application, though this is only possible if all necessary supplies are used. Glycemic monitoring can be performed through capillary sampling for self-monitoring of blood glucose (SMBG) and through the flash glucose monitoring system (FGMS) [7,11]. Regarding the application of insulin, it can be performed subcutaneously, using a syringe or pen, and can also be applied through the continuous infusion system, which mimics the function of the pancreas and is considered the best form of administration [7,12].

According to Ordinance No. 2538/2007 [13], insulin and supplies for glycemic monitoring must be available by the Unified Health System (Sistema Único de Saúde) in Brazil. However, access to supplies is not always easy, especially in a pandemic situation, when there may be delays in the supply of Basic Health Units. Therefore, sometimes the patient needs to buy the supplies, which is an unachievable criterion for many. Holman et al. [14] observed in England a significant association between the number of deaths related to COVID-19 in people with T1DM and having a lower purchasing power.

Although the glycemic control of patients with T1DM has been analyzed in several studies during the COVID-19 pandemic, there is a lack of studies that analyze the continued use of CC by these patients during the pandemic. Therefore, the purpose of this study was to analyze the sociodemographic and treatment factors associated with adherence to the CC strategy by adults with T1DM while social distancing during the COVID-19 pandemic in Brazil.

## 2. Materials and Methods

### 2.1. Type of Study

This is an observational, analytical study, carried out in July 2020, during the period of social distancing in Brazil. Social distancing was a strategy used for all sectors of society, ensuring that people remained in their homes during the validity of the decree of this measure by local administrators [15].

Data collection was performed using an online form (Google^®^ Forms platform), in the opinion survey format, according to Resolution N.510/2016 [16]. The questionnaire was published on social media networks directly to people who claimed to have T1DM in their social media biography (Whatsapp^®^, Instagram^®^ and Facebook^®^). To reinforce the idea that the research was aimed at people with T1DM, the research link was sent along with a message that specified that the research was directed at people with T1DM and over 18 years of age.

### 2.2. Participants

Convenience sampling was carried out with adults with T1DM, who consented to participate in the research voluntarily, anonymously, and without any financial compensation, and signed the informed consent form (ICF). The data of people who marked any different any different information from the abovementioned inclusion criteria were excluded. Thus, the participants who marked any of the following alternatives were excluded and the survey closed automatically: being legally responsible for a minor with diabetes; being a child/adolescent with Type 1 diabetes; having Type 2 diabetes; having diabetes of another type (gestational, LADA, MODY, etc.); having diabetes, but not knowing which type; or not fitting into any of the options presented. Participants who reported being younger than 18 years old and older than 59 years old, as well as adults with T1DM who did not complete the survey or who did not agree with the ICF, were also excluded. In that regard, 576 people responded to the questionnaire, but only 472 met the inclusion criteria.

### 2.3. Instrument

An online form was used, developed by the study researchers, with 18 objective questions, divided into five axes:

(a) Sociodemographic: age range (was recorded in numbers only, for example: 22), sex, characterization of the city and housing district in which one lived, and schooling level.
(b) Financial situation: family income in minimum wage (MW), considering the minimum wage value in the year of 2020 in Brazil (1.045 BRL); family income during the pandemic; and receiving financial emergency aid offered by the federal government to some people in social vulnerability.
(c) Supplies’ acquisition (considering the period 30 days prior to answering the form): equipment used for insulin administration, and equipment used for blood glucose monitoring.
(d) Food habits (considering the period 30 days prior to answering the form) and CC: increased consumption of sweet foods; increased consumption of ultra-processed foods, considering frozen ready-made foods such as nuggets, pizza, or cheese bread; fruit consumption (appropriate, for the consumption of 2 or 3 servings; and inappropriate, for the consumption of less than 2 servings or more than 3 servings); vegetable consumption (appropriate, when consumption was equal or more than 2 servings; and inappropriate, when consumption was less than 2 servings); number of daily meals; increase in cooking habits; and CC (did not know what it is; had heard about CC, but did not know how to perform it; did know how to perform CC, but was not engaged in it; stopped performing it during this period of social distancing; performed it more frequently than before social distancing; performed it at the same frequency as before social distancing; or performed it less frequency than before social distancing).
(e) Social distancing: type (total, was not leaving home to any activity; partial, left home only to buy food and medicines; did not engage in social distancing because of work commitments; did not engage in social distancing because they did not agree with it; engaged in social distancing for family reasons, but did not agree with it); and time one thought it was feasible to remain in social distancing.

### 2.4. Data Analysis

For statistical analysis, the SPSS^®^ software (v.24) was used. Descriptive results were expressed in absolute frequency and proportion. In the analytical step, the variable “CC Practice” was grouped into yes or no, regardless of the reason. Therefore, individuals who marked the options: (a) I do not know what CC is; (b) I have heard about it, but I do not know how to perform CC; (c) I know how to perform it, but I do not engage in CC; and (d) I stopped CC in this period of isolation, were classified as “No”, referring to not performing CC, and individuals who marked the options: (e) I perform CC more frequently than before isolation; (f) I perform CC at the same frequency as before isolation; and (g) I perform CC less frequently than before isolation, were classified as “Yes”, referring to engaging in CC. The Chi-square test of independence with adjusted residual analysis was applied to test associations between “CC Practice” and sociodemographic, economic variables, insulin administration, blood glucose measurement, social distancing, and eating.

Prior to the logistic regression analysis, the absence of collinearity between the study variables was observed through linear regression, observing tolerance and variance inflation factor values, all being greater than 0.1 and less than 10, respectively. Finally, a binomial logistic regression analysis was performed, composed of the dependent variables “did CC or not did CC” and the independent variables consumption (or not) of ultra-processed foods and cooking (or not) during social distancing. The final model was able to predict 62.7% of the carbohydrate counting practice in the studied sample.

A statistical significance level of *p* < 0.05 was considered.

## 3. Results

Among the 472 participants, most were between 25 and 44 years old (56.99%), were female (86.00%), had higher education (78.39%). A total of 37.71% reported that they performed CC at the same frequency as before social distancing; 20.13% reported performing CC more frequently than before; 18.01% reported that they had heard about CC, but did not know how to perform it; 13.77% reported that they did know how to perform CC, but they were not engaged in it; 5.08% reported that they performed CC less frequently than before; 2.97% reported that they stopped performing CC during social distancing and only 2.33% did not know what CC was.

Lack of higher education was associated with no performing CC (*p* < 0.000). There was an association between performing CC and living in the state capital (*p* = 0.027), as well as living in upper class neighborhoods (*p* = 0.025). Living in the interior of the state (*p* = 0.027) was associated with not performing CC. Earning between 5 and 10 MW (*p* < 0.000) and not receiving financial emergency aid because they did not meet the criteria (*p* = 0.045) was also associated with performing CC. However, having a family income of between 1 and 2 MW (*p* < 0.000) and having received financial emergency aid (*p* = 0.045) was associated with not performing CC (Table 1).

CC execution was associated with using an insulin pump and using flash glucose monitoring system (FGMS) along with the glucometer. While for those using a syringe, or pen and syringe, and using only the glucometer for glycemic measurement, there was an association with not performing CC (*p* < 0.000). CC execution during isolation was associated with being in total social distancing (*p* = 0.026). However, being in partial social distancing (*p* = 0.026) and not being able to spend more than a month in isolation (*p* = 0.013) was associated with not performing CC (Table 2).

The majority of participants reported consuming the same number of sweets as before the pandemic (37.08%), equal or less amounts of ultra-processed foods (68.65%), having an inappropriate fruit consumption (57.42%), having an appropriate vegetable consumption (53.39%), having between three and four meals a day (47.67%), and cooking more than before social distancing (50.86%). 

Cooking more during the pandemic was associated with CC execution (*p* = 0.012). Not performing CC was also associated with having more than six meals a day (*p* = 0.015) and consuming more ultra-processed foods (*p* = 0.022) (Table 3). 

Maintaining or decreasing consumption of ultra-processed foods was a significant predictor of performing CC, given that those participants who maintained or reduced consumption of ultra-processed foods were 0.62 times more likely to perform CC. Furthermore, the habit of cooking during the pandemic was a potential factor to performing CC, given that those participants who cooked more were 1.67 times more likely to perform CC (Table 4).

## 4. Discussion

In the present study, we observed that a large percentage of the respondents were women, similar to what was observed in the study by Coroiu et al. [17], which aimed to describe indices of motivations for social distancing during the COVID-19 pandemic. In the study by De Vasconcelos et al. [18], a study focusing on adults with T1DM, it was also observed that being female was associated with regular physical exercise during social distancing. In this context, data from the Ministry of Health indicate that women have greater concerns and care for their health, in addition to attending the healthcare system more often [19]. Therefore, the hypothesis is suggested that because of this concern, women were taking social distancing measures more seriously compared to men.

It was possible to observe the association between not performing CC and not possessing higher education. To perform CC, it is necessary to make calculations that require good mathematical skills, which may explain the difficulty in adhering to this strategy for patients with a lower level of education [20]. In addition, it is hypothesized that having less education can make it difficult to understand the importance and execution of the method.

Associations was observed between performing CC and living in the state capital, living in upper class neighborhoods, having a high family income, and not receiving financial emergency aid. For the implementation of CC, it is necessary that the patient has the necessary supplies to make the glycemic measurement and calculation for the application of insulin regularly [7,11]. In Brazil, the supplies for self-monitoring of blood glucose (SMBG) provided by the public system may not reach the necessary or recommended quantities for adequate monitoring. As a result, some people need to pay for these treatment costs, often using a significant amount of family income for this purpose. Thus, people with lower purchasing power may end up using lower doses of medication or reducing the frequency of SMBG to save money [21].

It is assumed that there is difficulty in accessing supplies for residents of inland cities and that the offer by the Unified Health System may suffer irregularities. In addition to this, the high cost of supplies and financial impact on people with low income may also hamper the proper implementation of CC [21]. No studies were found that evaluated the supply offers by the Unified Health System during the COVID-19 pandemic in Brazil, thus, it is not known whether the distribution by the government was taking place on a regular basis, which could affect the management of the disease and adherence to CC. 

No studies were found that analyzed the relation between adherence to CC and glucose measurement technology during the pandemic. Nonetheless, in one study [22], it was observed that 82% of the patients with T1DM who used the continuous insulin infusion system reported the usage of the pump calculation functions, such as the bolus calculator; and that these functions eased the calculation of the insulin dose. In addition, in the same study, an association was shown between using the insulin pump and using FGMS, suggesting that this can assist in adherence to CC.

It is possible to observe that partially social distancing and considering oneself unable to stay a whole month in this condition were associated with not performing CC. Fortin et al. [22] observed an association between not feeling able to perform CC and having meals outside the home, and 32% of the participants affirmed that performing CC requires time and delays the beginning of the meals. Hence, spending more time at home may have facilitated the adherence to CC, as individuals could have more time to perform CC and seek information on the composition of the foods.

No studies were found that could assess the relationship between cooking habits and practicing CC. Nonetheless, it is suggested that due to the participants staying longer in their homes, the habit of preparing their own meals may have contributed to a better understanding of the meals’ composition, since they knew the amounts of each ingredient in each recipe prepared themselves, facilitating adherence to CC. 

It is possible that the consumption of a greater number of meals was associated with not practicing CC, because in order to calculate CC it is necessary to know how to identify all food containing carbohydrates and estimate the size of the portions, which is time-consuming and requires discipline [22]. Fortin et al. [22] observed that the difficulties in performing CC were in estimating the amount of carbohydrates before starting the meals and believing that CC complicates the treatment of diabetes.

Having consumed more ultra-processed foods was associated with not performing CC. This result contrasts with the study by Fortin et al. [22], where 20% of the participants admitted to frequently consuming processed foods due to the easy access to nutritional information through the label, making CC easier. On the other hand, a study [23] with 50 people with T1DM identified that 82% of the participants were interested in reading the nutritional information provided on the labels in order to perform CC. Thus, it is hypothesized that people who perform CC may improve the quality of the food they eat, because it is recommended that CC is performed in the context of healthy eating, where the consumption of ultra-processed foods should be avoided [7,8].

The present study has the limitations of not having been carried out using a validated questionnaire, due to the following factors: the lack of a validated questionnaire for people with T1DM that investigates such aspects; not having assessed the glycemic control of the participants, making it impossible to draw associations between the adherence to CC and the levels of glycemia; and because it is an online survey, which may have caused a bias in the population studied, including only those individuals with internet access. However, no studies were found that evaluated the factors associated with the practice of CC in participants with T1DM during the COVID-19 pandemic.

## 5. Conclusions

The majority of people with T1DM continued performing or increased the frequency of CC performance during the period of social distancing. However, there are still people who do not know how to perform CC, which suggests the need for investment in diabetes education. It was also observed that having a higher family income, living in state capitals and in upper class neighborhoods, using insulin pumps and FGMS, practicing total social distancing, cooking more, and having lower consumption of ultra-processed foods was associated with greater adherence to the practice of CC. Furthermore, maintaining or decreasing the consumption of ultra-processed foods and the habit of cooking during the pandemic were a predictor factor of performing CC.

This research demonstrated that CC could assist adhering to a healthy lifestyle even during the period of social distancing. It is reiterated that the need for the regular acquisition of supplies to perform the glycemic monitoring is a crucial factor for the implementation of CC in the routines of these patients.

## Figures and Tables

**Table 1:** Association between CC execution and sociodemographic and economic data of people with T1DM during social distancing in Brazil, July 2020.

	Carbohydrate Counting *n* (%)		*p*-Value *
	Yes	No	
*Schooling*			
Higher education	247 (52.33)	123 (26.06)	<0.000 †
No higher education	50 (10.60) (−)	52 (11.01) (+)	
*City*			
State Capital	130 (27.54) (+)	57 (12.08) (−)	0.027 †
Metropolitan Region	68 (14.41)	40 (8.47)	
State inland	99 (20.97) (−)	78 (16.53) (+)	
*District*			
Favela or community	4 (0.85)	6 (1.27)	0.025 †
Periphery	46 (9.75)	33 (6.99)	
Middle Class	164 (34.75)	96 (20.34)	
Upper class	57 (12.08) (+)	16 (3.39) (−)	
Rural Area	11 (2.33)	9 (1.91)	
None of the alternatives	15 (3.17)	15 (3.17)	
*Family Income*			
<1 MW	8 (1.69)	11 (2.33)	<0.000 †
≥1 and <3 MW	66 (13.98) (−)	68 (14.41) (+)	
≥3 and <5 MW	94 (19.92)	59 (12.50)	
≥5 and <10 MW	78 (16.53) (+)	25 (5.30) (−)	
≥10 and <20 MW	38 (8.05) (+)	8 (1.69) (−)	
≥20 MW	13 (2.75)	4 (0.85)	
*Financial Emergency Aid*			
Yes	104 (22.04) (−)	81 (17.16) (+)	0.045 †
No, but no one met the criteria	173 (36.65) (+)	82 (17.37) (−)	
No, even requesting for financial aid and meeting the criteria	20 (4.24)	12 (2.54)	

* Chi-square. † Statistical Significance; Residue Analysis: (+) Significant Association (−) Negative Significant Association; MW = Minimum Wage.

**Table 2:** Association between adherence to the CC strategy and insulin administration, blood glucose measurement, and social distance from individuals with T1DM during social distancing in Brazil, July 2020.

	Carbohydrate Counting *n* (%)		*p*-Value *
	Yes	No	
*Insulin administration*			
Insulin pump	88 (18.64) (+)	6 (1.27) (−)	<0.000 †
Pen	167 (35.38)	100 (21.19)	
Syringe	6 (1.27) (−)	35 (7.42) (+)	
Both (pen and syringe)	36 (7.63) (−)	34 (7.20) (+)	
*Blood glucose monitoring*			
Glucometer	190 (40.25) (−)	154 (32.63) (+)	<0.000 †
FGMS	12 (2.54)	6 (1.27)	
FGMS and glucometer	94 (19.92) (+)	12 (2.54) (−)	
I do not perform blood glucose monitoring	1 (0.21)	3 (0.64)	
*Type of social distancing*			
Total	68 (14.41) (+)	21 (4.45) (−)	0.026 †
Partial	184 (38.98) (−)	126 (26.69) (+)	
No distancing because they needed to work	41 (8.69)	26 (5.51)	
No distancing because they did not agree	0 (0.00)	1 (0.21)	
Distancing for family reasons, despite not agreeing	4 (0.85)	1 (0.21)	
*Time one thought it was feasible to remain in social distancing*			
Would not be able to stay a whole month in this condition	25 (5.30) (−)	31 (6.57) (+)	0.013 †
Would be able to stay between 1 and 2 months	45 (9.53)	17 (3.60)	
Would be able to stay more than 2 months	15 (3.18)	9 (1.90)	
Was willing to stay as long as necessary to face the pandemic	212 (44.92)	118 (25.00)	

* Chi-square. † Statistical significance; Residual analysis: (+) Significant association (−) Negative significant association; FGMS = flash glucose monitoring system.

**Table 3:** Association between CC execution and alimentation of people with T1DM during social distancing in Brazil, July 2020.

	Carbohydrate Counting *n* (%)		*p*-Value *
	Yes	No	
*Sweets Consumption*			
Much higher	45 (9.53)	31 (6.57)	0.830
Slightly higher	96 (20.34)	54 (11.44)	
Same as before social distancing	109 (23.10)	66 (13.98)	
Decreased	47 (9.96)	24 (5.08)	
*Ultra-processed food consumption*			
Increased	82 (17.37) (−)	66 (13.98) (+)	0.022 †
Maintained or Decreased	215 (45.55) (+)	109 (23.10) (−)	
*Fruit Consumption*			
Adequate	129 (27.33)	72 (15.25)	0.627
Inadequate	168 (35.60)	103 (21.82)	
*Vegetable Consumption*			
Adequate	159 (33.69)	93 (19.70)	0.934
Inadequate	138 (29.24)	82 (17.37)	
*Number of Meals*			
More than 6	11 (2.33) (−)	18 (3.81) (+)	0.015 †
Between 5–6	132 (27.97)	77 (16.31)	
Between 3–4	150 (31.78)	75 (15.89)	
Between 1–2	4 (0.85)	5 (1.06)	
*Cooking Habit*			
Did not know how to cook	15 (3.18)	14 (2.97)	0.012 †
Did not like to cook, someone else was cooking	29 (6.14)	24 (5.08)	
Were cooking as much as before	71 (15.04)	40 (8.47)	
Were cooking less than before	17 (3.60) (−)	22 (4.66) (+)	
Were cooking more than before	165 (34.96) (+)	75 (15.90) (−)	

* Chi-square. † Statistical Significance; Residual Analysis: (+) Significant Association; (−) Negative Significance Association.

**Table 4:** Binomial logistic regression between adherence to carbohydrate counting, consumption of ultra-processed foods, and cooking habits by adults with type 1 diabetes mellitus during social distancing due to the COVID-19 pandemic in Brazil, July 2020.

	B	S.E.	Wald	df	Sig.	OR (Odds Ratio) *	95% C.I. for EXP(B)	
							Lower	Upper
Ultra-processed food consumption	−0.468	0.205	5.238	1	0.022	0.626	0.419	0.935
Cooking Habit	0.516	0.193	7.107	1	0.008	1.675	1.146	2.447
Constant	0.428	0.149	8.239	1	0.004	1.534		

* OR-odds ratio (OR = eβ); Binomial logistic regression. Dependent variable: adherence to carbohydrate counting; independent variables: consumption of ultra-processed foods and cooking habits.

## Data Availability

The data presented in this study are available on request from the corresponding author. The data are not publicly available due to the research was conducted through an online form that allows access to other data not used in this article.
